# Sub PPM Detection of NO_2_ Using Strontium Doped Bismuth Ferrite Nanostructures

**DOI:** 10.3390/mi14030644

**Published:** 2023-03-12

**Authors:** David John Dmonte, Aman Bhardwaj, Michael Wilhelm, Thomas Fischer, Ivo Kuřitka, Sanjay Mathur

**Affiliations:** 1 Centre of Polymer Systems, Tomas Bata University in Zlin, Tr. Tomase Bati 5678, 760 01 Zlín, Czech Republic; dmonte@utb.cz; 2 Institute of Inorganic Chemistry, University of Cologne, Greinstr. 6, 50939 Cologne, Germany

**Keywords:** gas sensing, bismuth ferrite, ppb

## Abstract

The present work investigates the NO_2_ sensing properties of acceptor-doped ferrite perovskite nanostructures. The Sr-doped BiFeO_3_ nanostructures were synthesized by a salt precursor-based modified pechini method and characterized by X-ray diffraction (XRD), scanning electron microscopy (SEM), and X-ray photoelectron spectroscopy (XPS). The synthesized materials were drop coated to fabricate chemoresistive gas sensors, delivering a maximum sensitivity of 5.2 towards 2 ppm NO_2_ at 260 °C. The recorded values of response and recovery time are 95 s and 280 s, respectively. The sensor based on Bi_0.8_Sr_0.2_FeO_3–δ_ (BSFO) that was operated was shown to have a LOD (limit of detection) as low as 200 ppb. The sensor proved to be promising for repeatability and selectivity measurements, indicating that the Sr doping Bismuth ferrite could be a potentially competitive material for sensing applications. A relevant gas-sensing mechanism is also proposed based on the surface adsorption and reaction behavior of the material.

## 1. Introduction

Nitrogen dioxide (NO_2_), one of the most evident air pollutants, tends to have a very pungent, biting scent. This well-known air pollutant affects lung function, increasing the risk of respiratory inflammations [1]. The World Health Organization (WHO) has recently emphasized a more stringent annual mean limit of ambient NO_2_, reducing the exposure limit from 40 µg/m^3^ to 10 µg/m^3^ [2]. It is shown not just to contribute asthmatic symptoms but is connected to significant morbidity in asthma patients [3]. Its presence in the atmosphere is key as a precursor in the formation of tropospheric ozone, atmospheric aerosols, and acid rain [4]. In sectors that use industrial plants, environmental monitoring, and air quality assurance for clinical practices and healthcare, accurate toxic gas detection at low concentrations is essential to the growth in these fields. Even lower concentrations of NO_X_ at parts per billion (ppb) are required, as they can be used to detect lung tissue infections [5]. Many types of sensors exist, such as amperometric and electrochemical, but their curbed LOD or higher operating temperature is shown to be limiting for extremely sensitive applications [6,7]. One of the many driving forces, apart from the many inherent advantages, are metal oxide semiconductor-based sensors, which have been the most researched type of gas sensing among all the other types [8]. The wide range of metal oxides with diverse morphologies, including NiO, In_2_O_3_, WO_3_, ZnO, and SnO_2_, have been explored for gas-sensing applications [9,10,11,12,13]. ZnO is the most widely used sensitive material for the detection of NO_2_, thanks to its wide band gap (~3.37 eV), non-toxicity, robust chemical, and electrical stability [14]. However, various other nanomaterials such as spinel metal oxides are being actively researched as sensing materials.

In general, spinel structures are well-researched in the field of gas storage, separation, and battery catalysis. Their use has been reviewed in literature and their special structure has sparked enough interest in the gas-sensing field [15]. Perovskite oxides have become particularly attractive for several applications, such as photocatalysts [16,17]. In the high vacancy region, oxygen can be diffused into the octahedra [BO_6_], which correlates with hole density, increasing conductive pathways. They can provide microstructural and morphological stability, tunable with a variety of dopants due to their flexibility in substitution of A and/or B position to further improve their exquisite electronic structure and electron mobility [18,19]. Doping enables gas sensors towards NO_2_ based on such perovskite structure have been a new direction of research. Yoshiteru et al. have used a trimetallic oxides SmFe_1–x_Co_x_O_3_ (x = 0−1.0) prepared by pyrolysis was used in a conductometric sensor [20]. Another interesting research direction was the preparation of a highly selective organic–inorganic heterojunction, which used a halide perovskite and InGaZnO to detect low concentrations of NO_2_.by Mani et al. [21]. A more sustainable approach was taken by Juan et al. to investigate the gas-sensing mechanism of two lead free perovskites Cs_3_CuBr_5_ and Cs_2_AgBiBr_6_ supported on Graphene for ultrasensitive detection of 500 ppb NO_2_ [22].

In this review by Rahul et al., they mentioned bismuth ferrite-based perovskite gas sensors showing excellent stability for over 1 year, although the response value and operating temperature could be improved. They also mention that there are more publications employing this material, which may be because they can now operate at lower temperatures and may soon become commercially significant [23]. Therefore, it is essential to research this material. On taking the electron structure of surface Fe ions (i.e., the relatively high number of coordinately unsaturated d-orbitals due to the lack of oxygen bonding) into consideration, bismuth ferrite nanoparticles are expected to have excellent gas sensitivity because the dangling bonds can provide more geometrically and electrically suitable locations for molecular chemisorption in comparison to a simple bimetallic oxide, which is very essential for enhancing its gas-sensing property. This can be increased much further by raising the grain surface-to-volume ratio, and doping will have significant impacts on the sensing property [24]. Similar research was published by Xu H, but with a low doping percentage showing the material to be sensitive to ethanol and acetone gas [25]. It is also well-established that the modifications induced in the properties of Sr-doped BiFeO_3_ change with an increase in the dopant [26]. Hussain et al. showed that bismuth ferrite changes its phase from rhombohedral (R3c) to pseudo-cubic (Pm-3m) with a Sr doping amount of 25%. With Sr doping, oxygen vacancies are generated in the crystal lattice which changes the electrical properties of the bismuth ferrite, possibly due to the change of Fe coordination from octahedral to tetrahedral [27]. Bismuth ferrite, in its pristine form, is shown to sense NO_2_ and, upon further doping with palladium, increases the potential barrier on the surface. This results in the increment of the resistance of the sensor material, thereby improving response and recovery times, as shown by Shivaji et al. [28]. Another research using a similar perovskite BaTiO_3_ shows that doping with Strontium also increases electron density and thereby induces strong polarization on the surface. It is shown to facilitate NO_2_ and NH_3_ reacting with positively charged (δ+) O and H atoms on the surface. Note that the response towards the former is two times higher than the latter [29]. Therefore, as explained and pointed out from the literature review and previous studies above, the increase in Strontium doping in BiFeO_3_ would be very suitable for the application of gas sensing, as the increased distortions and newly created oxygen vacancies can be very vital for sensing oxidizing gases, such as NO_2_ in low concentrations.

In this study, based on research [30,31], we have investigated the gas-sensing properties of BiFeO_3_ (BFO) and Sr doped BiFeO_3_ (BSFO) towards sensitivity and selectivity for NO_2_. The prepared materials were characterized, then further used in sensors. These sensors were developed using a heater substrate following a standard procedure, after which it was subjected to preconditioning. The focus of this study is the materials that were prepared to be used as sensors in gas-sensing measurements for selectivity, sensitivity, and repeatability for very low concentrations. 

## 2. Materials and Methods

### 2.1. Preparation of Bi_1−x_Sr_x_FeO_3_ (x = 0, 0.20) Nanopowder

Bi_1−x_Sr_x_FeO_3_ (x = 0, 0.20) was synthesized by a modified pechini method [32]. Bi(NO_3_)_3_.5H_2_O (Thermo Fisher Scientific Inc., United States) was dissolved in diluted nitric acid. Fe(NO_3_)_3_.9H_2_O and Sr(NO_3_)_2_ were gradually added to the previously described solution when the bismuth nitrate was completely dissolved. Subsequently, citric acid and then a polymerizing agent, ethylene glycol, was added in a molar ratio of 1:1:2. The above solution becomes a clear transparent sol. This solution was dried at 80 °C for 24 h to remove most of the water content, resulting in the formation of a gel. The gel is the result of transesterification between the metal–citrate complex and the polymer agent, which was then heat-treated at 700 °C for 4 h in air and cooled down to room temperature. This way, the BFO and BSFO nanomaterials were successfully synthesized.

### 2.2. Characterization

The powder sample phase was studied by powder X-ray diffraction (XRD). It was carried out using a Cu-Kα radiation as an electron source on a Rigaku D/max ULTIMA III diffractometer (Japan). The surface morphology and particle shape were investigated by field emission scanning electron microscope (FE-SEM) and elemental analysis was done by energy dispersive X-ray spectroscopy (EDX) using the scanning electron microscope Nova NanoSEM450 (FEI company). Additionally, the surface compositions and their corresponding element valences were characterized by X-ray photoelectron spectrum (XPS) at a pressure in the 10^−9^ mbar range, using an ESCA M-probe Spectrometer from Surface Science Instruments, equipped with a monochromatic Al Kα excitation source (λ = 8.33 Å). The survey spectra were measured with 0.5 eV resolution, and the step size of high-resolution spectra was set to 0.05 eV step size. The spectra were charge-corrected and binding energies (BE) were assigned using adventitious carbon C 1s line position. The correction (BE estimation) process has an associated error of at least ±0.1 eV up to 0.2 ± eV for insulating samples [33]. High-resolution spectra deconvoluted peaks were fitted with GL(30) line shapes and Shirley-type backgrounds. Utilizing CasaXPS software (Casa Software), spectral adjustments, and peak deconvolution were carried out.

### 2.3. Sensor Fabrication

The powder sample was made into a 10-weight % solution with α-terpinol. This solution was further dispersed from a micropipette, and 10 µL was dropped on the prefabricated sensor substrate 4 pin-TO39 from UST Umwelt sensor Technik GmbH. This sensor was then allowed to dry at 80 °C for 18 h to achieve a uniformly smooth surface layer. This simple construction allowed us to the test the limit of the material’s sensitivity rather than other factors such as thickness, etc. The sensor was then sintered at 600 °C for 2 h before starting measurements for gas sensing. Three sensors of each material were prepared for further measurements.

### 2.4. Gas-Sensing System

**Figure 1:**
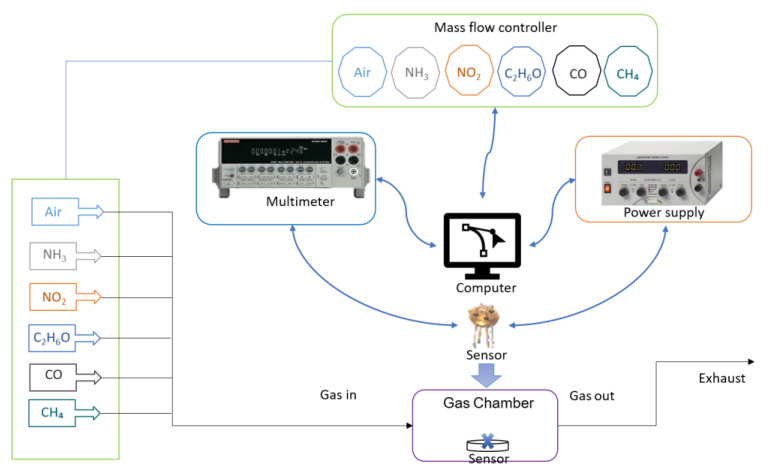
Schematic of gas-sensing system.

Gas-sensing measurements were investigated in a custom setup using an airtight chamber of about 250 mL with provision for gas injection and exhaust. This schematic is shown in Figure 1, and the desired concentration (in ppb and ppm) was achieved by mixing the analyte gas flow with synthetic air gas flow using two mass flow controllers (Aera FC-7700C) simultaneously. To rule out any surface interactions with moisture, the background environment was produced using a dry air gas cylinder (Air Products and Chemicals, Inc., certified for 0% humidity). The certified gas cylinders of target gases (NH_3_, NO_2_, CO, CH_4_, and C_2_H_6_O) were used for dilution in the measurements. The resistance in exposure to air and analyte mixes was continuously measured from the sensor using a Keithley K2700 data acquisition system. A calibration curve between voltage and temperature was prepared for accurate heating. The power source meter used was model ES-PS 3065-03B from Elektro Automatik, and it was used to power the platinum heater in sensor substrate TO-39. In Figure 1, the blue arrows represent the flow of data between the components of the system. The black arrows represent the gas flow through the chamber.

Every system and unit in this setup were controlled by custom programmed LabVIEW program to run and obtain experimental data. The gas-sensing setup is described in the supplementary document (Figure S1). The sensitivity to the responses was calculated by the following formula.

(1) $$S=\frac{Ra-Rg}{Ra}$$

(2) $$S=\frac{Rg-Ra}{Ra}$$

Equation (1) is for reducing gases and Equation (2) is for oxidizing gases. *R_g_* and *R_a_* stand for the resistance of the sensor in the presence of the analyte and the resistance of the sensor in synthetic air. The amount of time needed for the sensor to saturate to 90% of the total resistance is what is meant by T_90_ response and T_90_ recovery time [34,35,36].

## 3. Results and Discussion

### 3.1. Physical Properties

**Figure 2:**
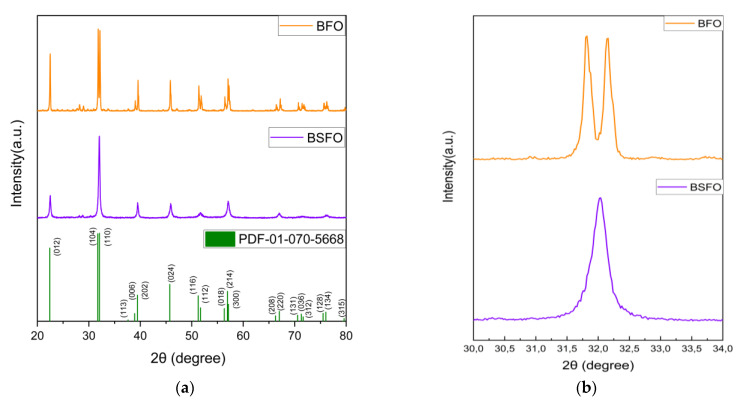
XRD patterns of (**a**) pure BFO and BSFO samples. (**b**) Peak merging of BSFO.

The XRD patterns in Figure 2a of the BFO match the peaks of powder diffraction file no: 01-070-5668, which belongs to the R3c space group with a rhombohedral structure. The main diffraction peaks at 2θ (2-theta) of 22.4, 31.72, 37.61, 38.91, 39.45, 45.72, and 51.26 well-matched the (0 1 2), (1 0 4), (1 1 0), (1 1 3), (0 0 6), (2 0 2), and (0 2 4) crystal planes [37]. The nanomaterial displayed almost identical peaks with no variation, and their crystal structures were well-indexed. The replacement of the Bi^3+^ in the BSFO spectra typically leads to some of the diffraction peaks disappearing, attributed to the relatively greater size of the Sr^2+^ (0.112 nm) as controverting to Bi^3+^ (0.103 nm), and to the difference in the size of crystals [25]. Figure 2b shows the enlarged part of the graph between 31° and 34°, which shows that in BSFO the doubly split peaks merge totally to form a single peak. This further confirms the presence of our doping and similar phase transformation behavior has been discussed by Hussain at al [27]. The crystallite size is given by Debye Scherrer equation = kλ/B cos θ. The value of k is set to 0.94 as we consider an approximately spherical shape and λ is set to 1.54 Å. The mean crystallite size is calculated for the peaks above in BFO to be 34 nm, and for BSFO it is 24 nm. They are also in close agreement with the SEM results.

**Figure 3:**
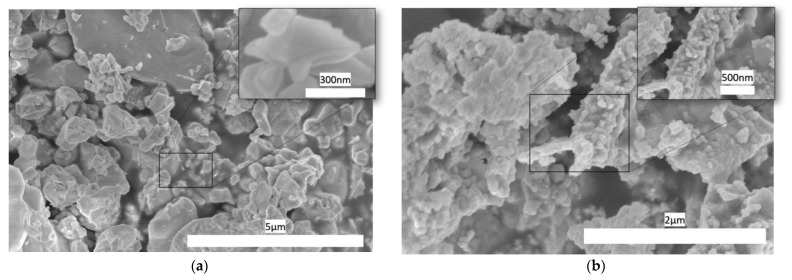
SEM images of (**a**) BFO flakes and (**b**) BSFO nanoparticles.

Scanning electron microscopy was used to confirm the materials’ nanocrystalline structure of BFO and BSFO. The SEM electrograph in Figure 3a,b show there are micrometer-sized primary flakes that are agglomerated with irregularly shaped nanoparticles on top of them. Their particle size is between 30 and 100 nanometers in size. The doping has contributed to the overall size by reducing the size of the particles overall. Strontium oxide has a greater melting point (2531 °C) than bismuth oxide (817 °C), which may be inhibiting grain development and causing the grain size to decrease [38]. EDX was also measured, and the values are listed below in the Table 1 for BFO and BSFO. The complete dataset is shown in the supplementary (Figures S2 and S3). They are in close agreement with the values published before, showing successful doping [26].

According to the elemental analysis, the atomic ratio of metals Bi: Fe in the original material is 4:1 which is quite far from the expected stoichiometry of BFO, indicating more Bi_2_O_3_ components in the structure. However, there is no such prominent crystalline phase in the X-ray diffractogram as one would expect if this were a bulk composition feature. Moreover, EDX analysis showed an atomic ratio of 1: 1. The probing depth of XPS may be estimated to be several nanometers at maximum, whereas the penetration depth of the e-beam in SEM is more than one micrometer. In the case of such a fine-grain material, EDX may be considered a bulk analysis method. Altogether, it means that the structure of the particles’ surface is iron deficient and is far from being stoichiometric, whereas the core of the particles consists of a BFO phase. It may also have an impact on the behavior of the semiconducting material, including a change between the band-bending behavior and bulk conductivity [39].

The compositions and chemical valence states of surface elements are characterized using X-ray photoelectron spectroscopy. The survey spectrum is displayed in Figures S4 and S5 in the supplementary document. The doping of the material by strontium induced significant changes in elemental ratios. According to XPS, the surface-related atomic concentrations are 5.4: 2.5: 1 for Bi: Fe: Sr, indicating the presence of Sr in the surface layer at the expense of Bi concentration. The bulk-sensitive EDX showed a ratio of 10.5: 4.2: 1 for Bi: Fe: Sr, which means that the ratios between Bi and Fe became comparable in bulk (2.5:1) and at the surface (2.2: 1). The results also indicate about two times higher strontium doping level at the surface than in the cores of the particles.

**Figure 4:**
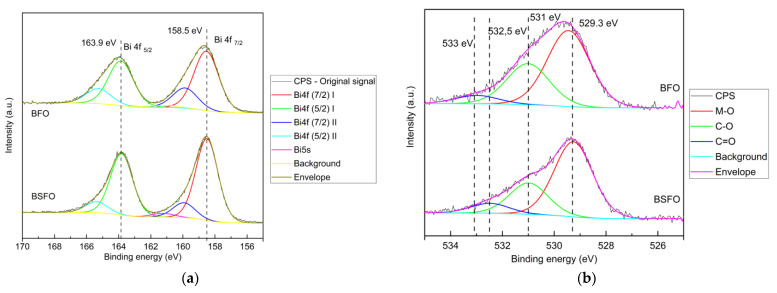
XPS high resolution scans of (**a**) Bi 4f of BFO and BSFO (**b**) O1s of BFO and BSFO.

The high-resolution XPS measurement is shown in Figure 3 and Figure 4. The peaks in the graph in Figure 4a at 158.5 eV and 163.9 eV are related to Bi 4f 7/2 and Bi 4f 5/2, respectively, indicating positively charged Bi in mixed oxide compounds [40]. A closer analysis shows two Bi 4f components testifying for two binding sites, one major component at 158.5 eV and 163.9 eV (red curve), which may be associated with Fe^3+^–O–Bi^3+^ environment in the crystalline lattice, and the minor component at 159.9 eV and 165.3 eV (blue curve), which may be associated with the Bi^3+^–O–Bi^3+^ environment in the crystalline lattice [41]. The ratio of corresponding areas is 3:1. The doping of BFO by 20% strontium caused a reasonable decrease in the relative signal intensity of the blue component to a ratio ca 6:1. It indicates the replacement of the Bi ions in the higher binding energy positions (blue) by Sr^2+^, while Bi ions in the lower binding energy positions (red) are not influenced. The spin-orbit splitting energy between Bi 4f 7/2 and Bi 4f 5/2 is 5.40 eV, a typical value for Bi. Graph (b) in Figure 4 shows the peak at 529.3 eV, which is a noted signature for skeletal oxygen ions O^2−^ in the lattice of crystalline oxides. The peak component at 531 eV is possibly due to the contribution of adsorbed carbon moieties containing the C-O group or due to hydroxyl groups, while the smallest component at 532.5 eV is associated with the carbonyl group (C=O) or carbonates. The relative signal intensity of the O 1s component at 531.0 eV decreased slightly with doping by strontium. However, this change might be due to the surface contamination moieties. These observations show a desirable change in the material surface structure due to the doping of Sr^2+^, as it could be very beneficial to the application of our aim in this work.

**Figure 5:**
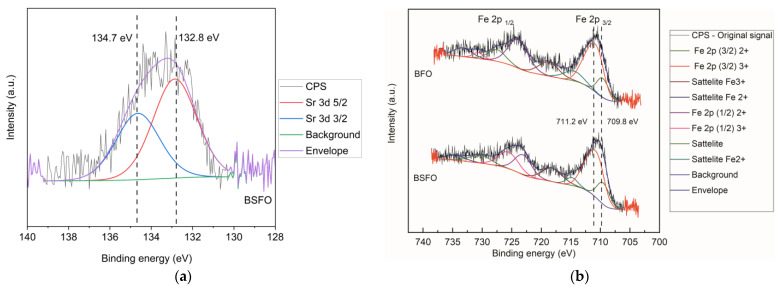
XPS high resolution scans. (**a**) Sr 3d of BSFO. (**b**) Fe 2p of BFO and BSFO.

To confirm the doping of Sr^2+^, it can be seen in Figure 5a that a faint peak with two components at 132.8 eV and 134.7 eV testifies the presence of the Sr 3d 5/2 and Sr 2d 3/2 and corresponding typical orbital splitting [25]. Figure 5b shows high-resolution spectra of both original and doped materials in the typical range for Fe 2p lines. The analysis of iron photoelectron spectra is extremely peculiar, and the Gupta and Sen (GS) multiplets are to be used as shown in [42]. We undertook the analysis using such a multiplet approach, however, we feel the quality of data as well as the material crystallinity and homogeneity is too low and the fitting procedure may be prone to inaccuracies or exaggerated interpretation. To avoid it, we adopted and revised the common view on the BFO nanomaterials identifying two main components in the materials as Fe^3+^ and Fe^2+^ [25,41]. Unlike in these references, we did not fit only a few peaks to the original data obtaining thus various Fe^3+^/Fe^2+^ ratios, but the whole GS multiplet was fitted.

On the other hand, we merged the fitted peaks for Fe^3+^ and Fe^2+^ multiplets to attain the desired level of clarity and simplicity, which is appropriate to the data quality. If one focuses on the Fe 2p 3/2 peak at 710.8 eV, two main components can be identified. One is centered at 709.8 eV and the other is centered at 711.2 eV. The component at lower binding energy can be associated with Fe^2+^ ions, whereas the one at higher binding energy can be associated with Fe^3+^ ions in the crystalline structure of the material. The ratio between the corresponding areas of the components is 5.0: 1 in favor of the higher binding energy sites. After doping, the ratio changes to 3.3: 1, but the higher binding energy sites still prevail. It was shown that the surface structure differs significantly from BFO stoichiometry, and it is not reasonable to expect only Bi^+3^, Fe^+3^, and O_3_^−2^ to form oxidation states. On the other hand, similarly to Bi, which is not expected in the form of Bi^2+^ at all, the consideration of Fe^3+^ and Fe^2+^ ions in well-defined states in the defect structure might be an exaggeration. Rather than that, we suppose the presence of iron in two different binding sites, distinguished by lower and higher binding energy depending on specific arrangements of oxygen, bismuth and iron atoms, and vacancies or impurities in their coordination spheres.

### 3.2. Gas-Sensing Properties

The sensing properties of both the BFO and the BSFO are evaluated by resistance change in the presence of either a reducing gas or oxidizing gas. The dynamic response of all the sensors was tested across a temperature range from 150 °C to 500 °C towards our target gas of NO_2_. After a preconditioning period of 2 h, a stable baseline was reached.

**Figure 6:**
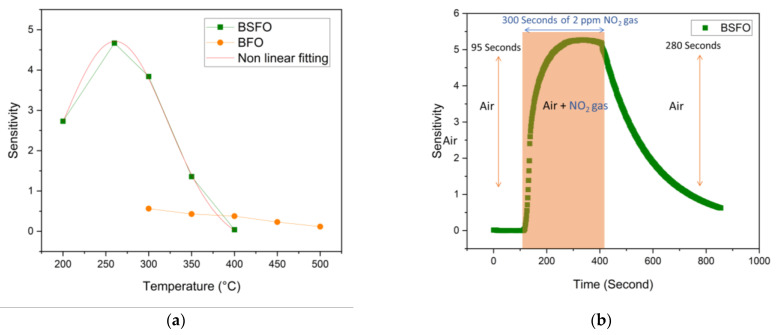
(**a**) Sensitivity chart of BSFO and BFO. (**b**) Response curve of BSFO at 260 °C.

The first basic test is to analyze the transients of dynamic sensing of both the sensors to the NO_2_ gas flow at all possible thermal kinetics by varying the temperature. It is understood that the response of the sensors to NO_2_ gas depends on the relative balance between the adsorption and the desorption of the NO_2_ gas molecules and the reaction with the adsorbed oxygen [43]. This adsorption lacks the required amount of energy in low temperatures, showing slow sensing kinetics. The increase in temperature provides more energy for this reaction, enhancing the response but more than adequately increasing the desorption. The BSFO sensor shows a response of 4.7 times for gas injection of 100 s 2 ppm NO_2_ to the baseline at the optimal temperature of 260 °C. This test is depicted in Figure 6a using a total flow rate of 200 sccm. The fitting of non-linear form using a gaussian function with an orthogonal distance regression as an iteration algorithm is graphed on the sensitivity chart to manifest the optimal range for the highest sensitivity. The reference undoped BFO sensor was very resistant to record any baseline resistance values below 300 °C. It can be seen that the drop in operating temperature might be attributed to the introduction of new energy levels caused by the Sr doping element as well as the nano-size effect [44]. This test is repeated with a small difference of extending the time of gas injection to 300 s to reach maximum saturation and calculate maximum response and recovery times for 2 ppm of NO_2_. The sensitivity reaches 5.2 times and is relatively stable. Irrespective of looking at the physicochemical results, we presume to have successfully created abundant oxygen vacancy defects in the perovskite lattice, which compensated the acceptor doping of Strontium atom at A sites. This means that the p-type conductive behavior of our material is enhanced and would help us have a sufficient measurable resistance at relatively low temperature. Additionally, the gas adsorption is higher at lower temperatures, and therefore we see an enhancement in the sensing response. This test is depicted in Figure 6b.

**Figure 7:**
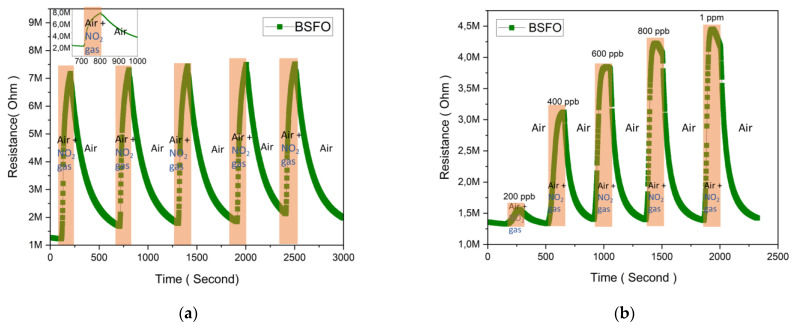
Transient response curves of BSFO. (**a**) Repeatability towards 2 ppm of NO_2_ at 260 °C. (**b**) Dynamic sensing characteristics to different concentration of NO_2_ at 260 °C.

The Figure 7a BSFO sensor was further tested for repeatability by using the NO_2_ gas, injection period of 100 s, and concentration of 2 ppm. It was seen that the sensor response stays constant over five cycles, showing that all sensors are reliable with consistent response and recovery times. The inset graphs show some level of saturation in this gas injection and show that fast kinetics is achieved. This is clearly due to the lattice distortion and phase transformation. The increase in carrier concentration and electron mobility leads to a substantially bigger hole accumulation layer at the surface of the nanoparticle, therefore improving the sensing performance [45]. Another test to evaluate the lowest detection limit of this sensor is by varying the concentration of the target gas. The sample is tested with the NO_2_ gas, which is injected into the gas chamber from 200 ppb to 1 ppm for a period of 150 s. As per our system design, a higher flow rate of 800 sccm was used to reach such low concentrations of the target gas. In Figure 7b this measurement is repeated until 1 ppm and the noticeable lowest response is from 200 ppb, but the response from 400 ppb, 600 ppb, 800 ppb, and 1 ppm are relatively higher and more consistent. Utilizing the lowest allotted settings in the mass flow controller to obtain our LOD value of 200 ppb electronically means that mechanical function should be taken into consideration because it is anticipated that the electronic control of the orifice will take more time to reconfigure itself. We do not, however, establish an exemption specifically for this situation. This could be one of a number of contributing causes to the relatively weaker response at the lowest concentration. Otherwise, in addition to the effects of doping, the increase in the surface area due to the nano-size grains also creates more active reaction sites with the NO_2_ gas molecules. Energetically, doping also reduces the band gap energy to amplify surface reactions, allowing for more feasible electronic reactions and better sensitivity, which we hold accountable for our sub ppm gas sensing [45].

**Figure 8:**
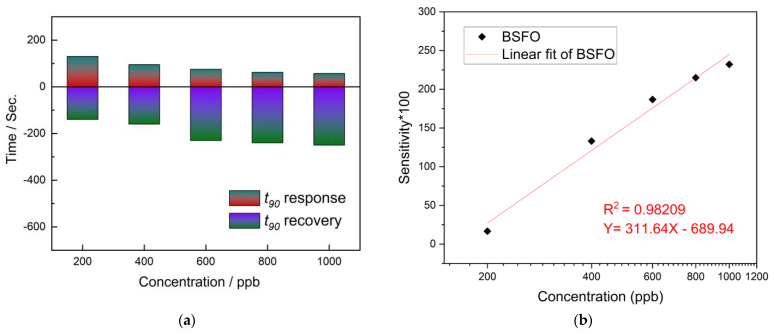
(**a**) Response/recovery dynamics. (**b**) Logarithmic dependency for the BSFO sensor.

The T90 response and T90 recovery are calculated and plotted in Figure 8a. The observable decreasing trend in T90 response time and increasing time in T90 recovery time is expected, as it explains the feasibility of the chemical reaction as the amount of target gas analyte increases in the system. A calibration curve is plotted in Figure 8b using the experimental data. Interference drawn from the graph shows that the calibration curve has a linear dependency between the sensitivity and logarithm of NO_2_ concentration under constant conditions. The exponential increase in the gas sensor response usually depicts a possibly cascading redox reaction which could be due to the influence of the dopant and thereby its new lower work function. The linear fit equation is Y = 311.64X − 689.94 with R^2^ = 0.98209. However, further studies are necessary to test its long-term stability.

**Figure 9:**
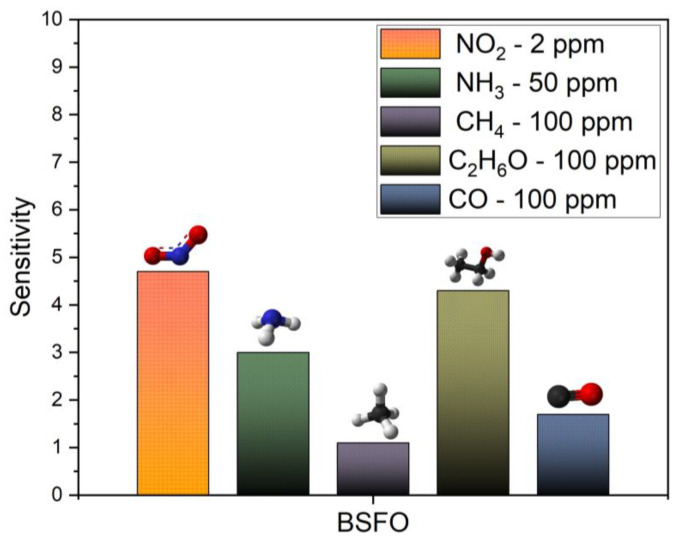
Cross-sensitivity test for BSFO at 260 °C.

Finally, the BSFO specimen was investigated for its cross-sensitivity properties in the same conditions and using the same setup with the operating temperature of 260 °C. The prepared sensors were tested in the same manner for a transient dynamic response with gas analyte injection for 100 s for sensitivity responses toward other gases. The concentrations are indicated in Figure 9, where the sensor response towards various gases is depicted with a bar graph. The sensitivity is calculated and the BSFO sensor shows the largest response of 4.7 towards our analyte gas NO_2_. This further confirms the selectivity of our target gas over other competing gases in separate environments. Most other gas analytes tested were of higher concentration due to system configuration and gas cylinder availability. It showed close to moderate response sensitivity, noting that the second largest response belongs to ethanol albeit in high concentration. The responses to NH_3_, CH_4_, and CO are minimum. The low gas response to other gases, excluding NO_2_, signifies its high specific adsorption capability towards NO_2_. A low gas response is credited to its low electron-withdrawing ability. This is presumably due to the weak adsorption/diffusion with the material surface. The reference undoped BFO sensor remained insulating at operating temperature.

### 3.3. Sensing Mechanism

Typically, the literature solely uses the adsorption-oxidation-desorption cycle to explain how these materials sense gases. In the current work, a possible gas-sensing mechanism was explained for sensing material BSFO. The gas-sensing mechanism for bismuth ferrite is known to be a p-type sensing behaviour and the doping, as shown above, changes the crystal structure, allowing for more vacancy defects. In the air, oxygen adsorbs on the sensor surface like reactions (1), (2), and (3).

(3) $$O_{2_{\left(ads\right)}}+e^{-}\leftarrow \to O_{2(ads)}^{-}$$

(4) $$O_{2(ads)}^{-}+e^{-}\leftarrow \to 2O_{\left(ads\right)}^{-}$$

(5) $$O_{\left(ads\right)}^{-}+e^{-}\leftarrow \to O_{\left(ads\right)}^{2-}$$

This reaction creates an electron-deficient surface by electron-‘trapping’ by the adsorbed oxygen during its reduction, increasing the electrical resistance of p-type oxides, henceforth widening the depletion region and the degree of band bending [46]. In the complex BSFO, the conductive surface as seen in SEM analysis consists of large micrometer particles and small nano-sized crystallites, enabling it to be more of a porous structure as well. This allows for both the surface bulk model and the nanocrystal model explained to be plausible [47]. However, after being oxidized by the exposure to a low concentration of NO_2_ gas, the resistance increases, only indicating a change in the surface conductivity, showing an opposing behavior that could be in contrast with the bulk conductivity of the material. The surface-adsorbed atomic oxygen species reacts with the high-affinity NO_2_ gas which reduces the electron concentration near the surface, giving rise to recombination with the holes, resulting in the resistance to increase. A plausible reason could be that NO_2_ gas diffuses onto the surface of the BSFO crystal grains, and oxygen ions react with NO_2_ in the following reversible process (4), (5), (6), and (7), forming a depletion region [48,49].

(6) $$NO_{2}+e^{-}\leftarrow \to NO_{2}^{-}$$

(7) $$NO_{2}+e^{-}\leftarrow \to NO+O^{-}$$

(8) $$2NO_{2}+O_{2}^{-}+e^{-}\leftarrow \to 2NO_{2}^{-}+O_{2}$$

(9) $$NO_{2}^{-}+O^{-}+2e^{-}\leftarrow \to NO+2O^{2-}$$

These changes which have occurred seemed to only affect our material surface only in the preset conditions such as low concentration, operating temperature, gas concentration, and humidity level of our measurement setup. This response observed follows the nanocrystal model. A similar mechanism was also reported by Aravind et al. [50]. NO_2_ is known to be a very oxidizing gas, and it is believed to be reduced in the gas-sensing process as described above. It is important to discuss the role of strontium dopant in the active sensing material. Based on our sensing results, we can presume that our polycrystalline material has several oxygen vacancies, allowing for a new and more efficient conduction mechanism. It is believed that the delocalized conjugated structure allows better electrical conductivity due to a site role of Sr^2+^ facilitating more electrons to the conduction band of the BSFO, consequently suppressing the grain boundary effects, interfacial, and dipolar polarization. This eventually leads to the enhanced gas-sensing performance [51]. The Sr-O bond has larger dissociation energy (426 kJ mol^−1^) compared to Fe-O (213 kJ mol^−1^), allowing for more oxygen bonding [52]. Therefore, we can say that the presence of strontium dopant functions as a catalyst reduces the work function required for the reversible reaction described above, thereby lowering the operating temperature and allowing for more binding sites. This explains the detection ability of low concentrations, in addition to the lower operating temperature, which is beneficial, as it means lower overall energy usage. Thus, due to the synergistic effect of both bismuth ferrite and strontium dopant, the sensing performance has greatly improved in comparison to pristine bismuth ferrite [53].

## 4. Conclusions

In this study, doped and undoped bismuth ferrite nanostructures were synthesized by sol–gel method and were evaluated for NO_2_ sensing properties in detail. The XRD spectra identified polycrystalline structure of both bismuth ferrite and Sr doped bismuth ferrite. The irregular-shaped nanoparticles and their size are shown to be between 30 nm and 100 nm, and the doping has further reduced the overall size of the nanoparticles. This composition for both BFO and BSFO seen in EDX is in correlation with the literature. The presence of expected elements of BFO and the dopant in BSFO are also confirmed in the XPS spectra. The as-prepared nanostructures, when used as gas-sensing materials for NO_2_, showed a highest sensitivity of 5.2. The gas concentration variation is shown with a linear fitting along sensitivity, where the limit of detection for BSFO is 200 ppb. The sensor exhibited fine repeatability, while the response and recovery times can be further improved by structural modification. These results confirm that the Sr-doped bismuth ferrite enables a lower working temperature, and higher responses are promising sensing materials for the development of low-cost, easily fabricated, stable, and high-performance NO_2_ gas sensors.

## Figures and Tables

**Table 1:** EDX shows the elemental analysis listed below.

	BFO			BSFO	
Element	Weight %	Atomic %	Element	Weight %	Atomic %
O	12.93	55.15	O	12.59	47.13
Bi	69.71	22.43	Bi	49.59	14.21
Fe	18.36	22.42	Fe	32.92	35.3
**-**	-	-	Sr	4.9	3.35

## Data Availability

Not applicable.

## Supplementary Materials

The following supporting information can be downloaded at: https://www.mdpi.com/article/10.3390/mi14030644/s1, Figure S1: This is gas sensing setup schematic; Figure S2: EDAX unprocessed data for BFO; Figure S3: EDAX unprocessed data for BSFO; Figure S4: Survey spectra for as prepared BFO material and C 1s high resolution spectra; Figure S5: Survey spectra for as prepared BSFO material and C 1s high resolution spectra; Figure S6: Raw data used for the sensitivity graphs in Figure 5a in the main draft BFO—response to NO_2_ gas BSFO response to NO_2_ gas.
