# Supplementary material for: Sub PPM Detection of NO2 Using Strontium Doped Bismuth Ferrite Nanostructures
Source: Micromachines (Basel). 2023 Mar 12;14(3):644. doi: 10.3390/mi14030644 (PMC10058199; doi:10.3390/mi14030644)
Supplement: Supplementary file 1 [file micromachines-14-00644-s001.zip › micromachines-2279578-supplementary.docx]

Supplementary Materials: Sub PPM Detection of NO_2_ Using Strontium Doped Bismuth Ferrite Nanostructures

David John Dmonte, Aman Bhardwaj, Michael Wilhelm, Thomas Fischer, Ivo Kuřitka
and Sanjay Mathur


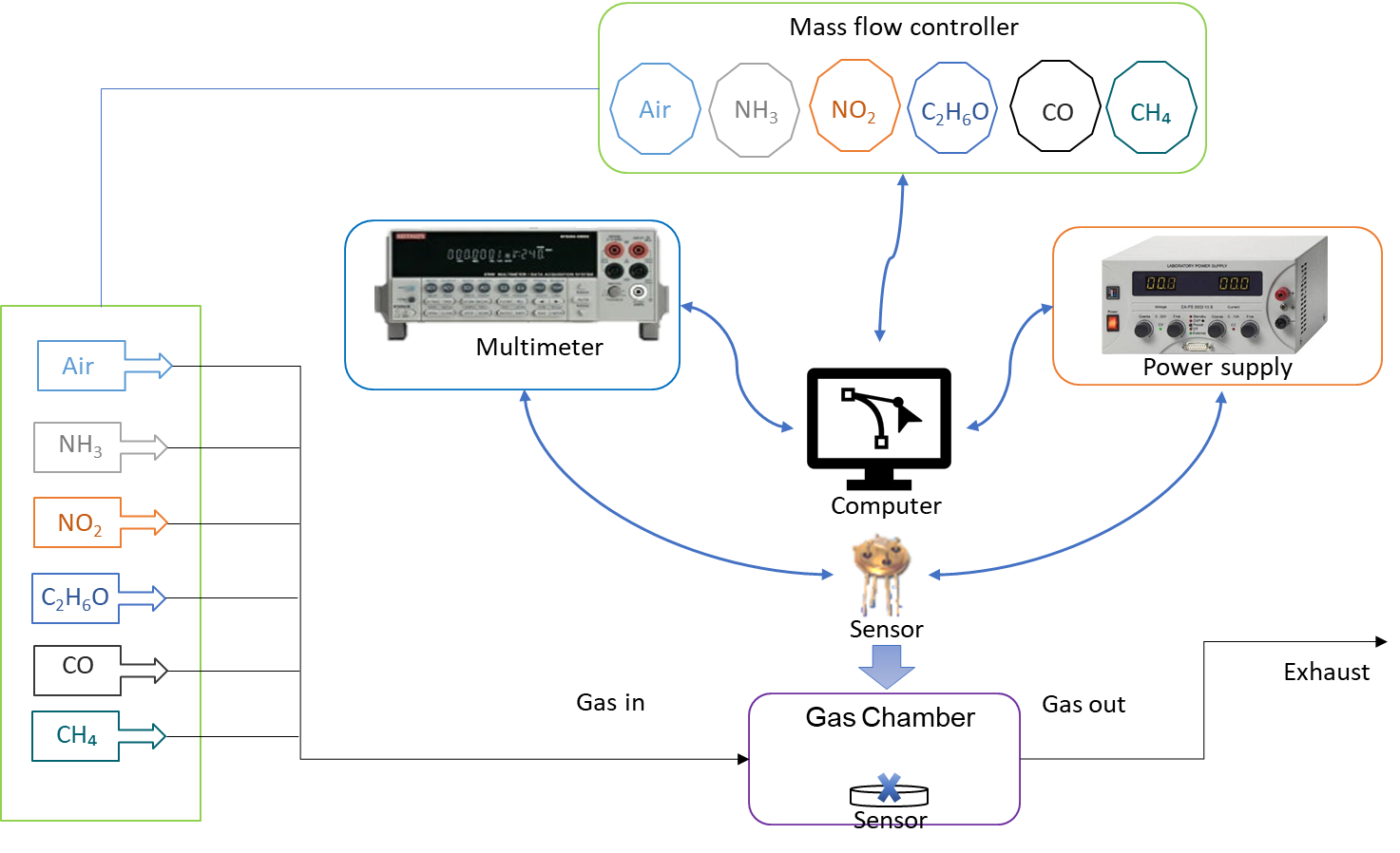


**Figure S1.** This is gas sensing setup schematic.

| 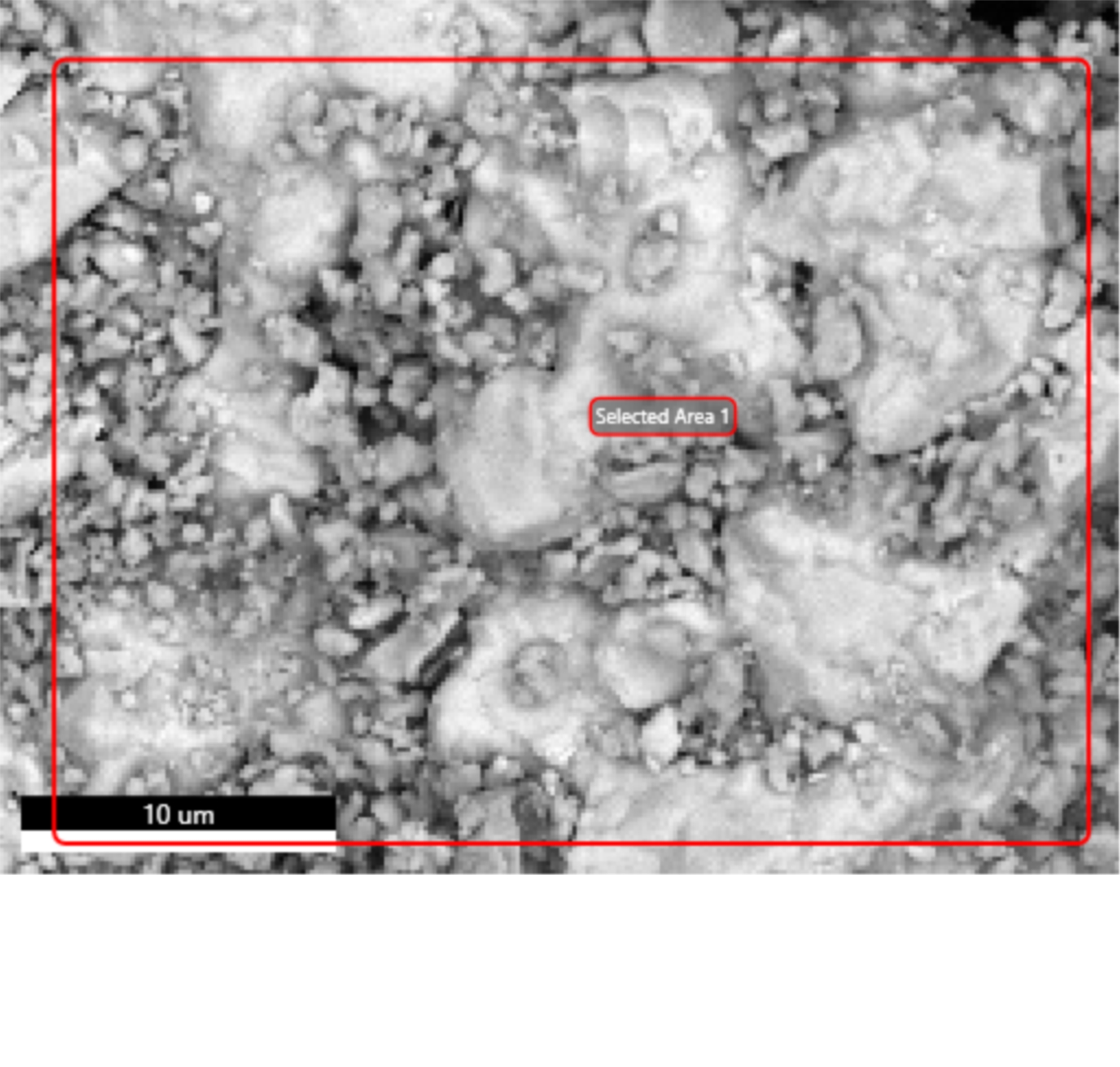 |
| --- |
|  |
| 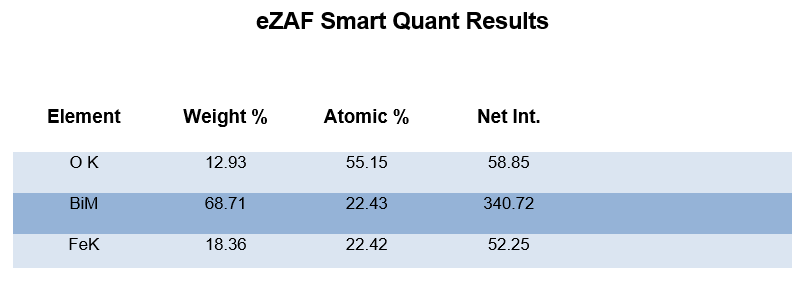 |
|  |
| 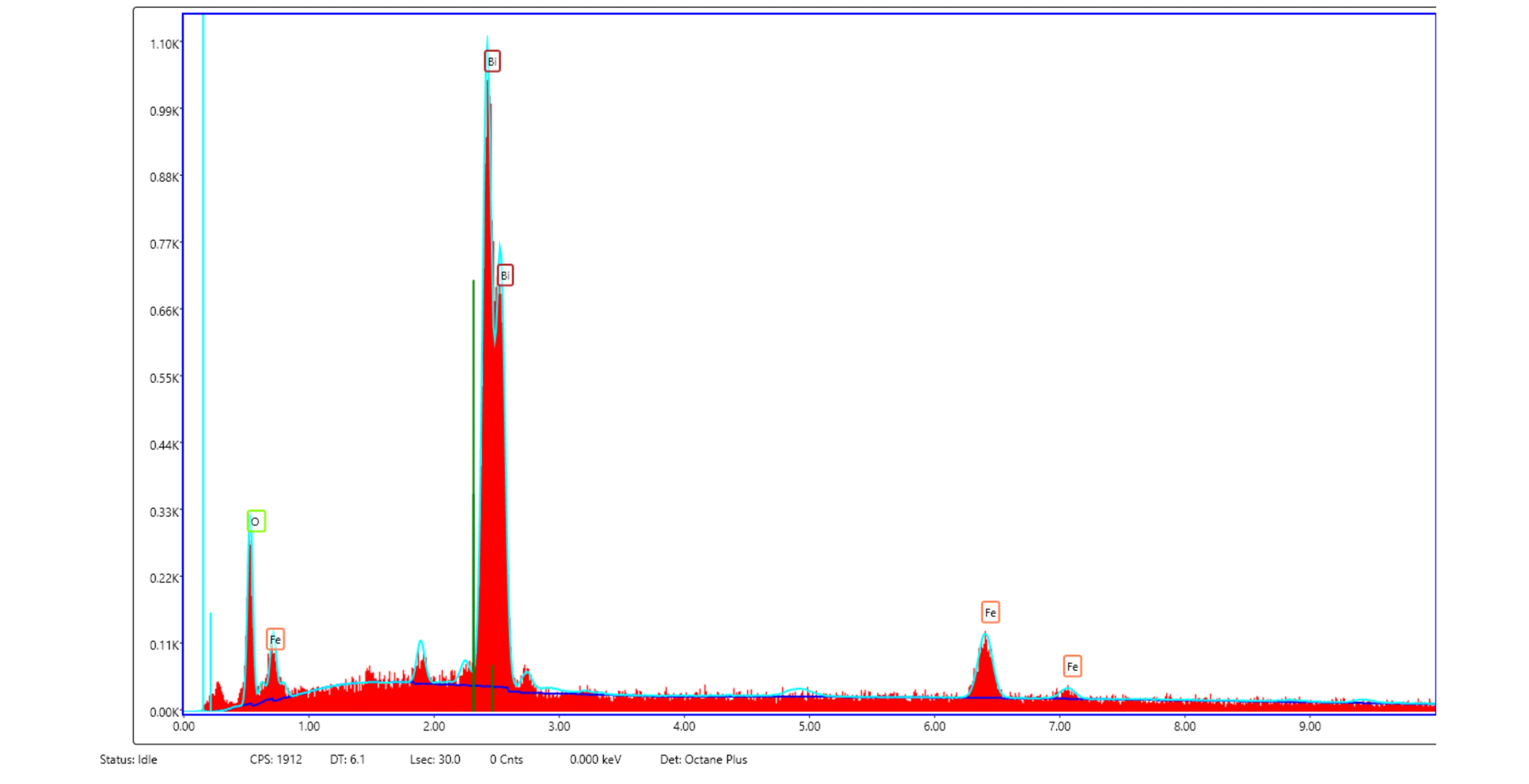 |
|  |

**Figure S2.** EDAX unprocessed data for (**a**) BFO, (**b**) scan area, (**c**) quants results

| **eZAF Smart Quant Results** | | | | | | | | | | | | | | |  | |  | |
| --- | --- | --- | --- | --- | --- | --- | --- | --- | --- | --- | --- | --- | --- | --- | --- | --- | --- | --- |
|  |  |  |  |  |  |  |  |  |  |  |  |  |  |  | |  | |  |
| **Element** | | **Weight %** | | | **Atomic %** | | | | **Net Int.** |  |  |  |  | |  | |  |  |
|  |  |  |  |  |  |  |  |  |  |  |  |  |  |  | |  | |  |
| O K | | 12.59 | | | 47.13 | | | | 86.5 |  | | | | |  | |  | |
|  | | | | | | | | | | | | | | |  | |  | |
| SrL | | 4.9 | | | 3.35 | | | | 42.96 |  | | | | |  | |  | |
|  | | | | | | | | | | | | | | |  | |  | |
| BiM | | 49.59 | | | 14.21 | | | | 328.35 |  | | | | |  | |  | |
| 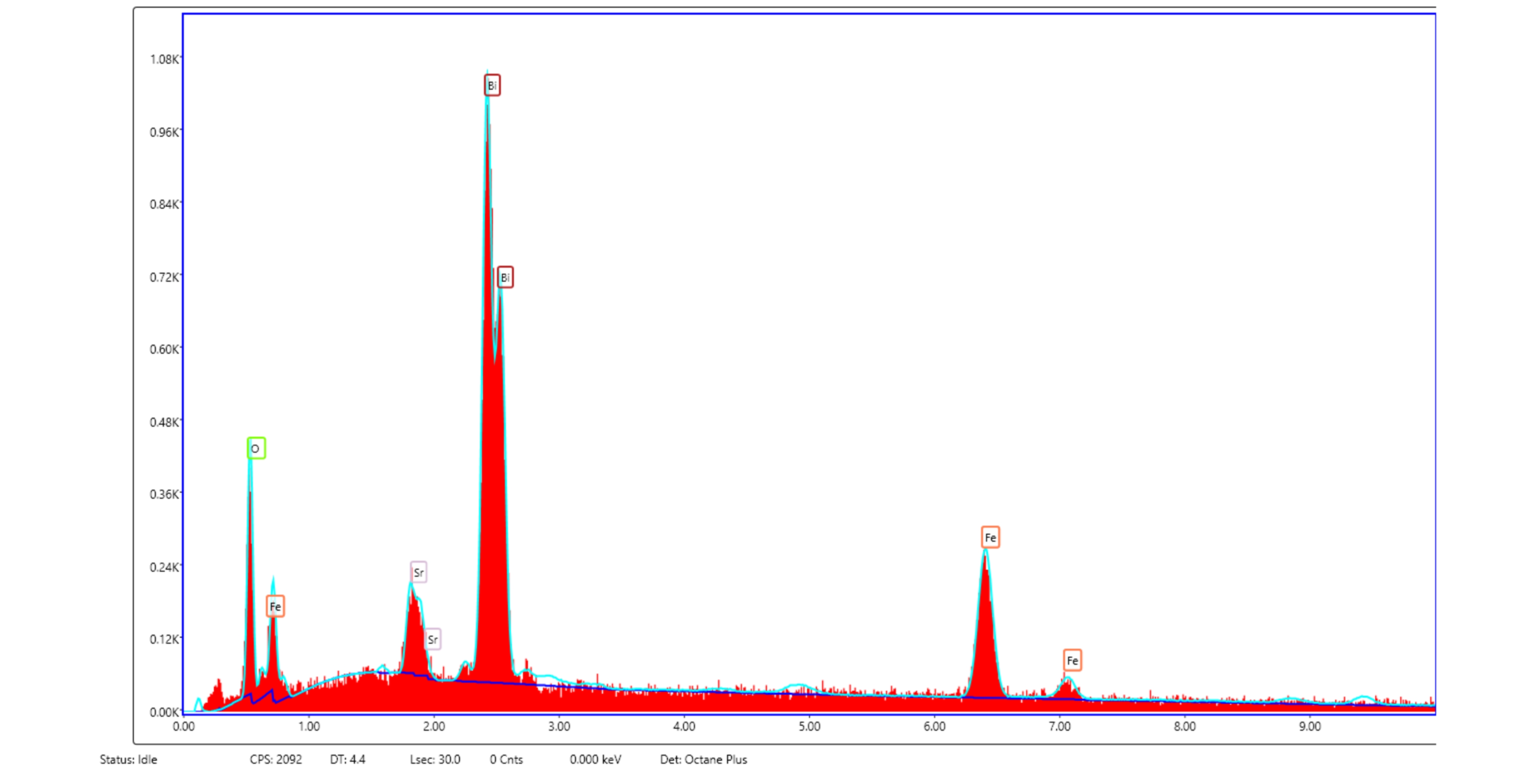 | | | | | | | | | | | | | | |  | |  | |
| FeK | | 32.92 | | | 35.3 | | | | 127.81 |  | | | | |  | |  | |


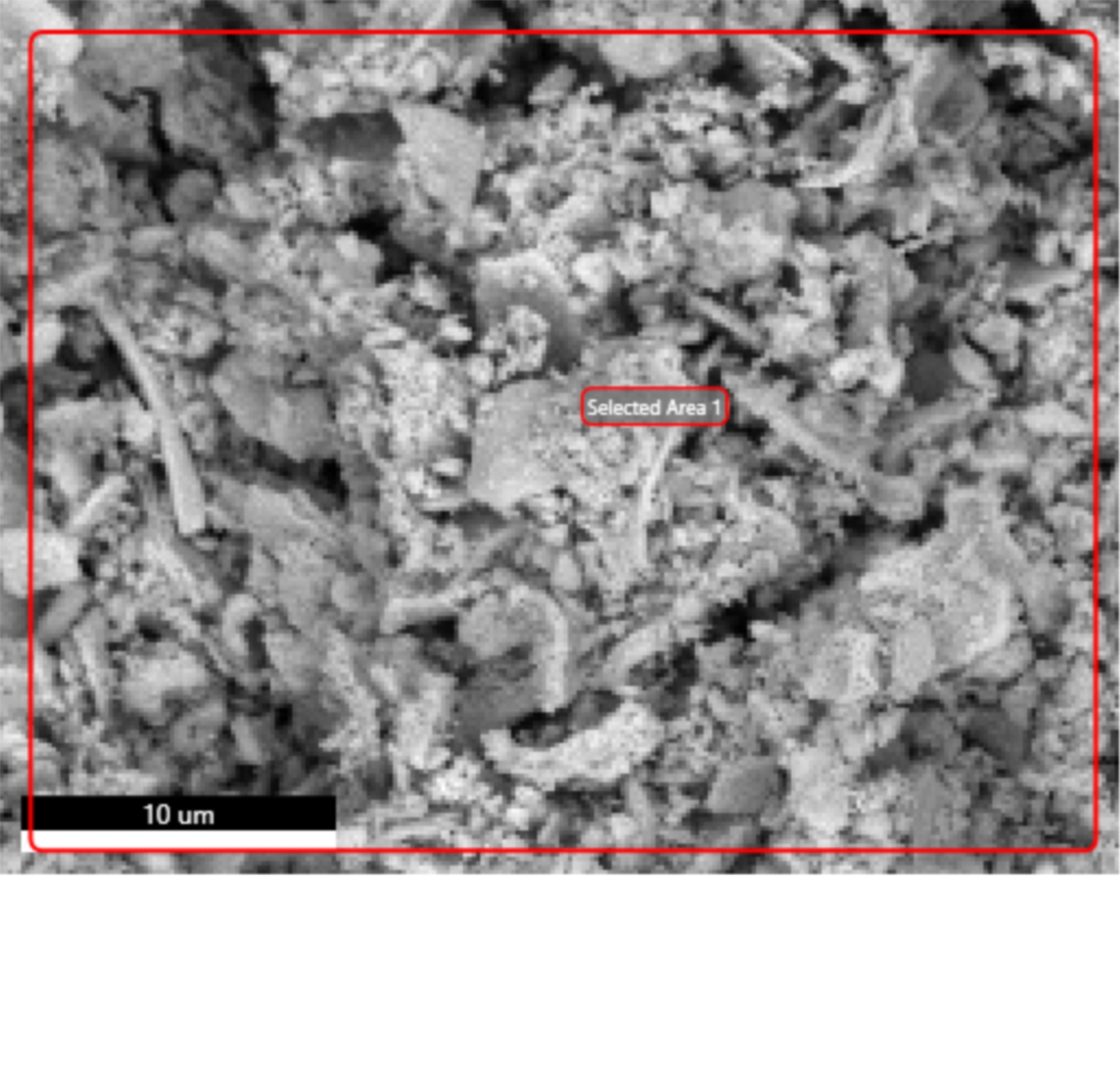


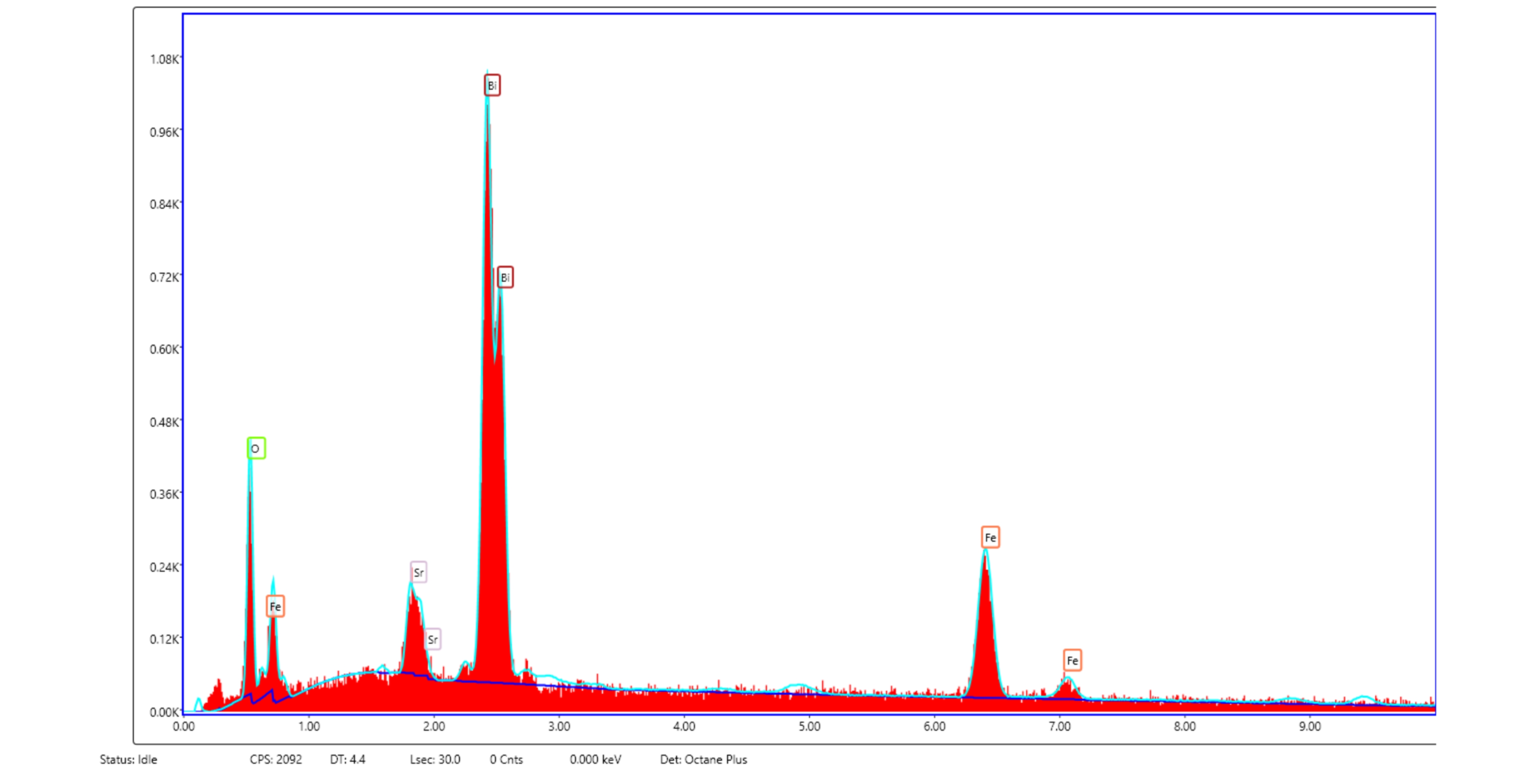


**Figure S3.** EDAX unprocessed data for BSFO


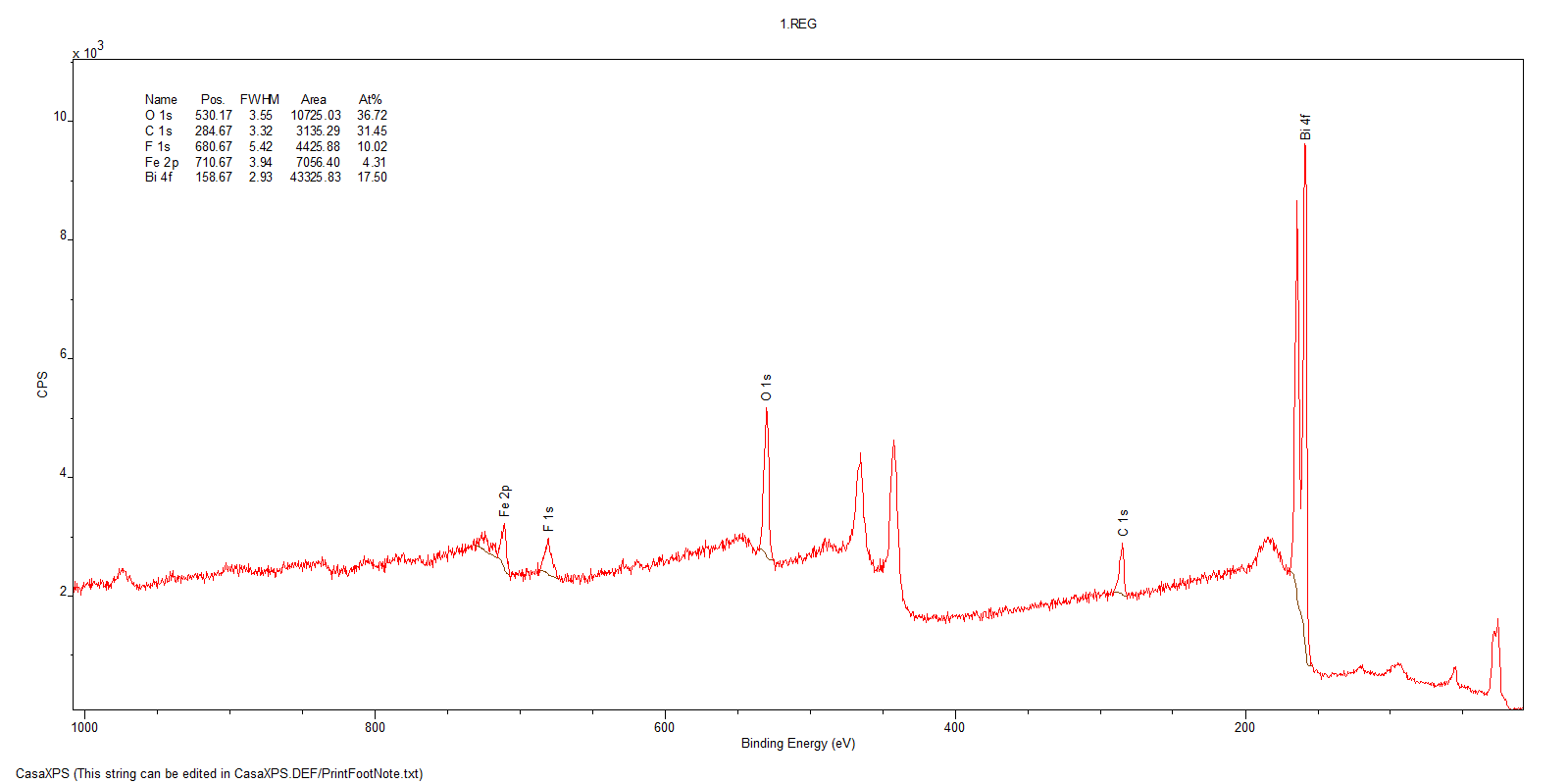

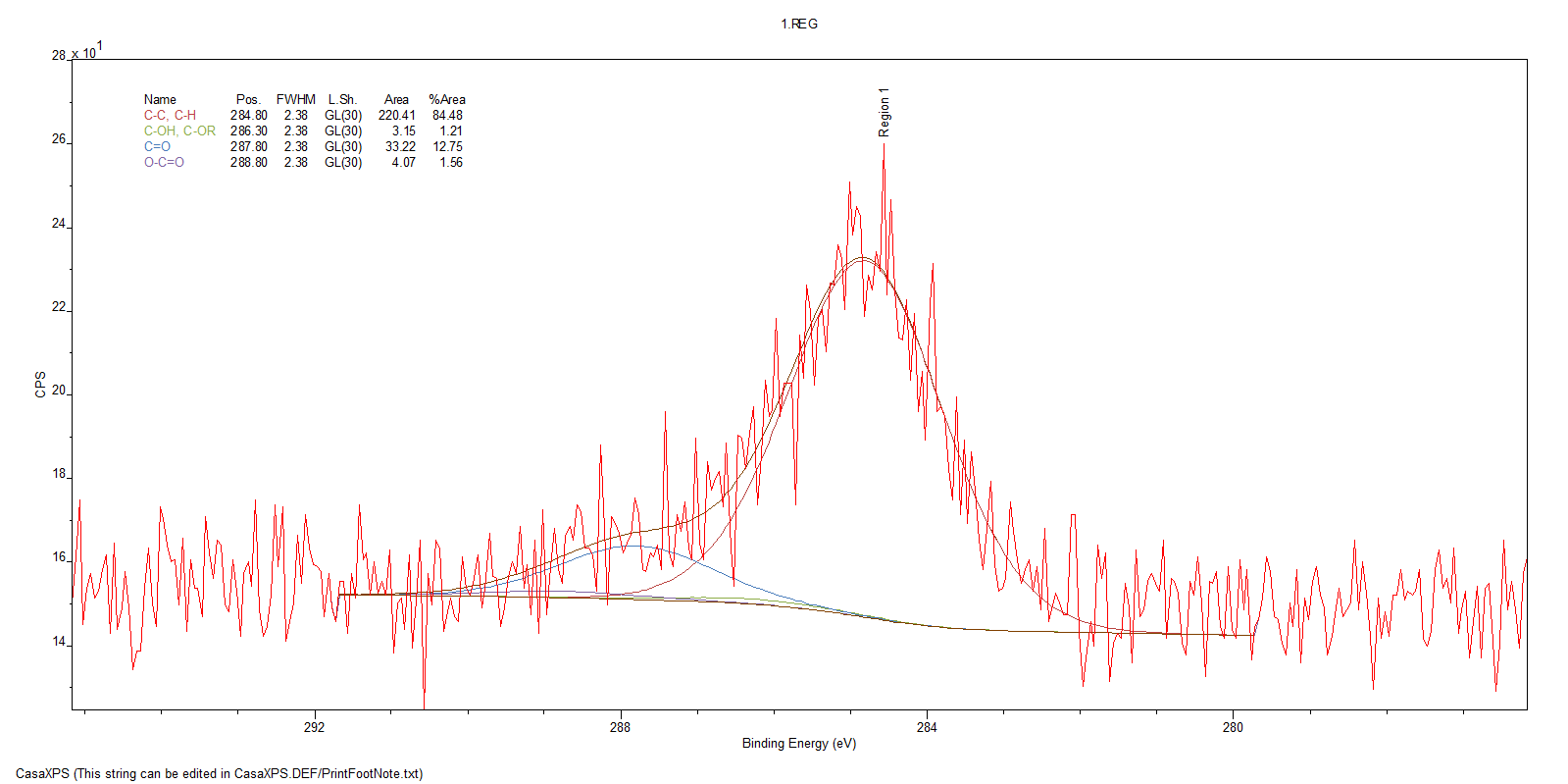


**Figure S4.** Survey spectra for as prepared BFO material (**above**) and C 1s high resolution spectra (**below**)

There seems to be a fluorine contamination in the survey spectra above, due to operator handling. However, it can be ignored as, it is not present in any other spectra and is therefore not present in our sensor. Besides confirmation of all expected elements, a nonnegligible presence of adventitious carbon was registered. It may come from organic compounds used in synthesis and from handling the samples in an ambient atmosphere.


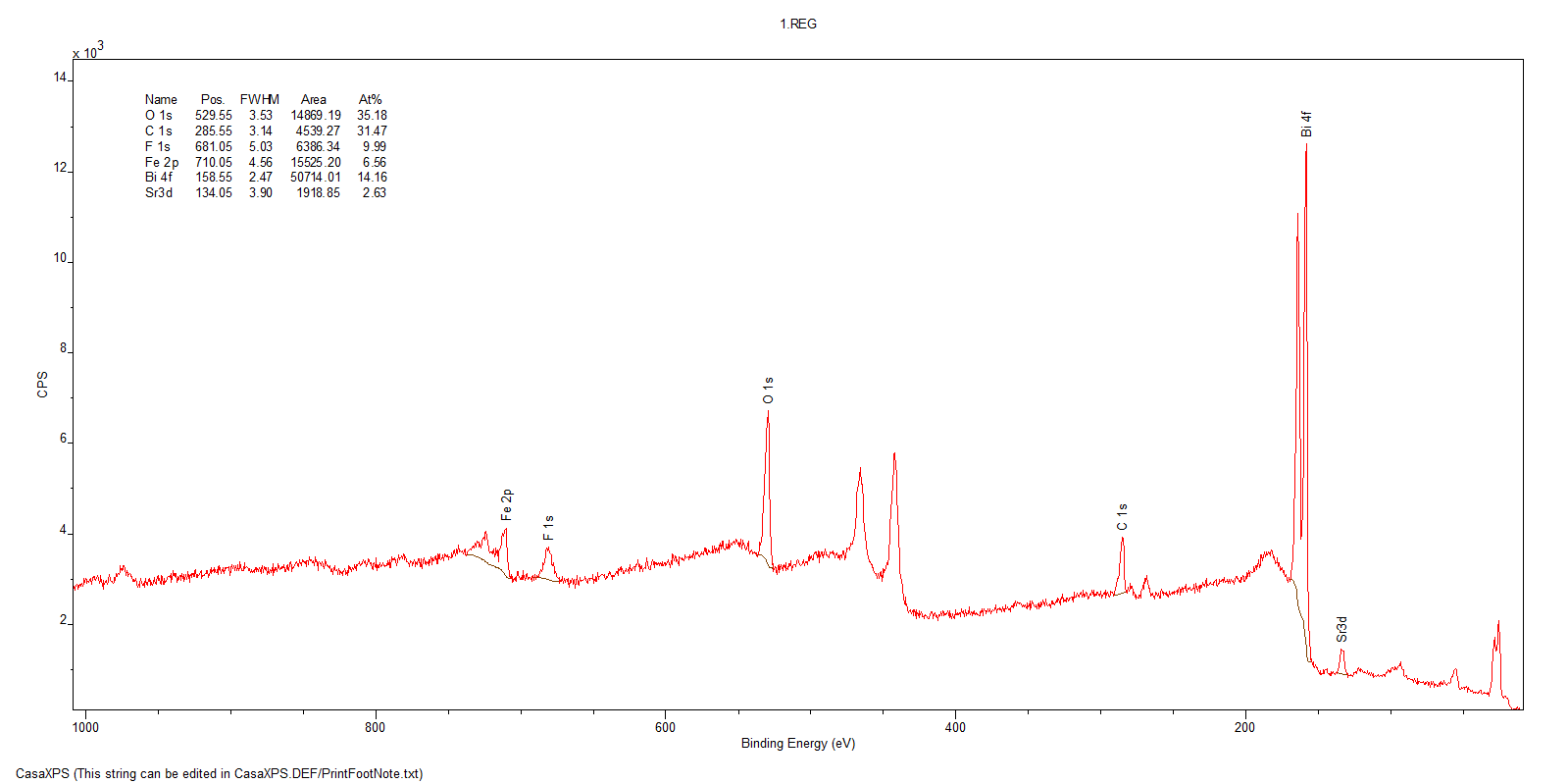


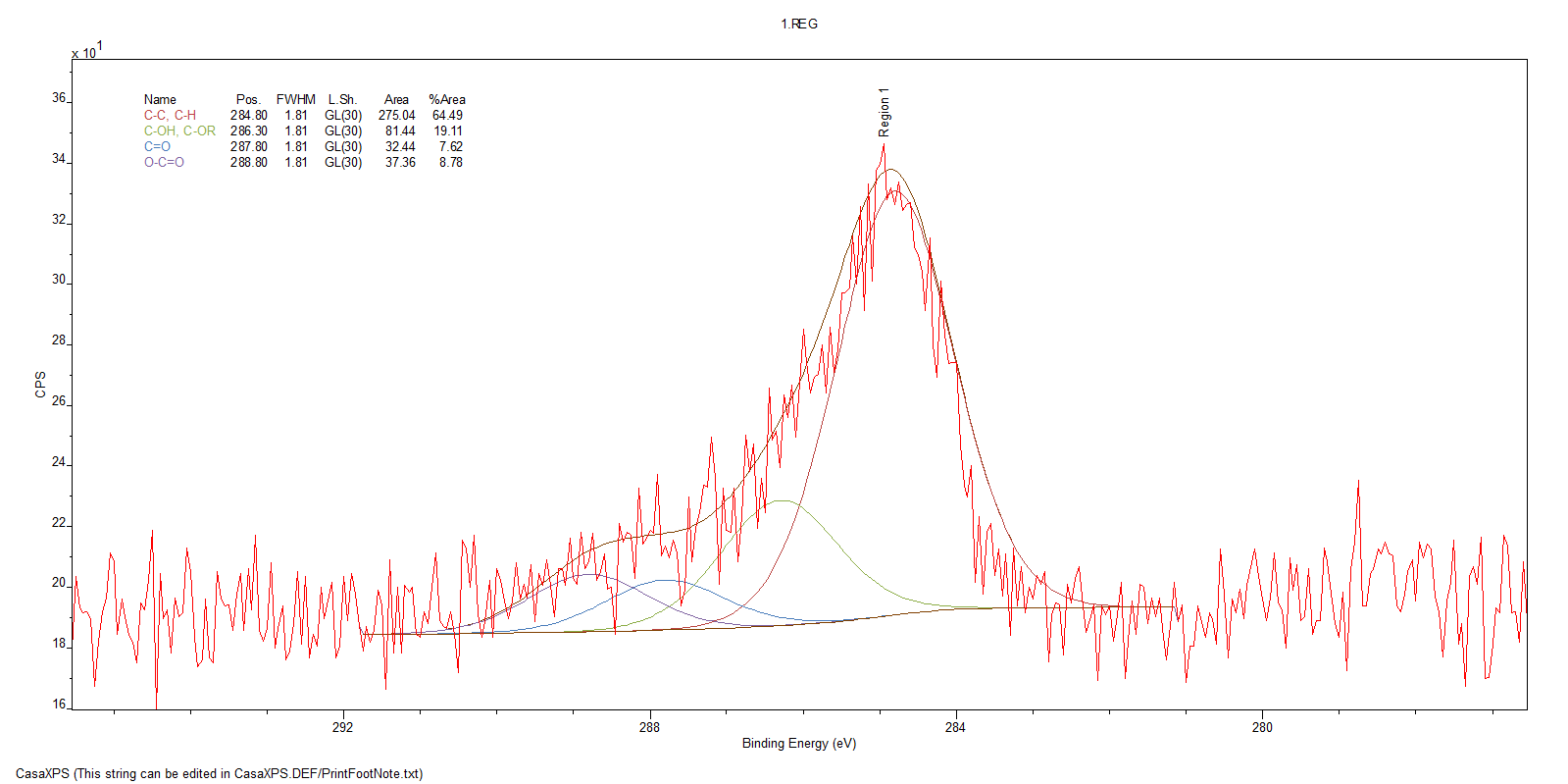


**Figure S5.** Survey spectra for as prepared BSFO material (**above**) and C 1s high resolution spectra(**below**).

Due to operator handling, there appears to be fluorine contamination in the survey spectrum above. It may be disregarded, though, as it doesn't appear in any other spectrum and is consequently absent from our sensor. In addition to the confirmed existence of all predicted elements, a miniscule bit of adventitious carbon was detected. It could result from handling the samples in an ambient environment and from organic substances employed in the synthesis.

**
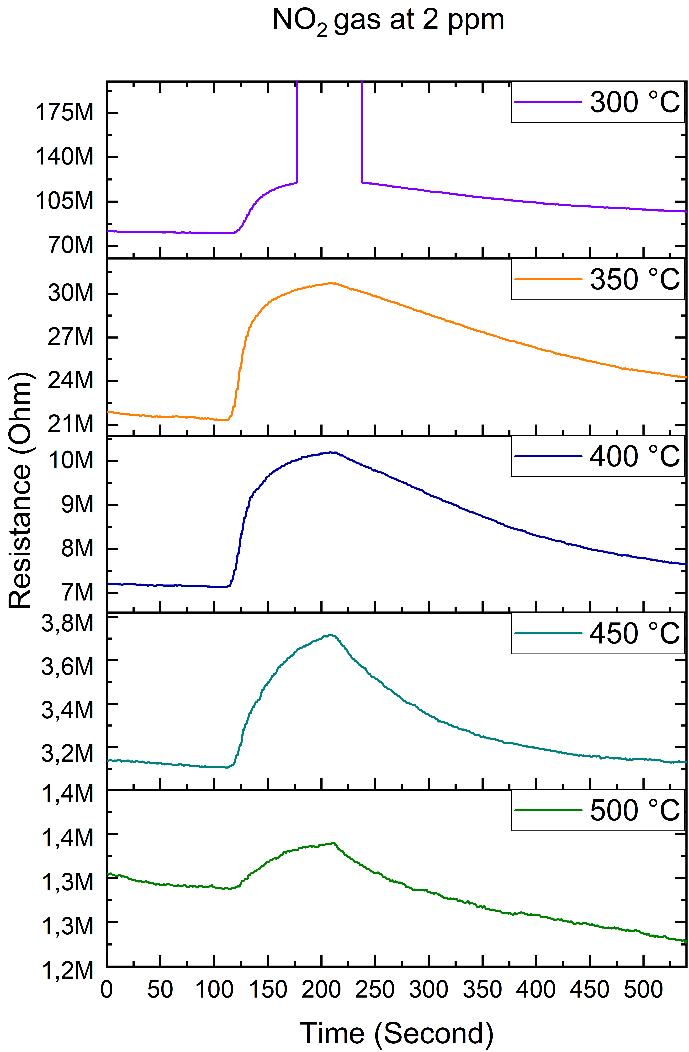

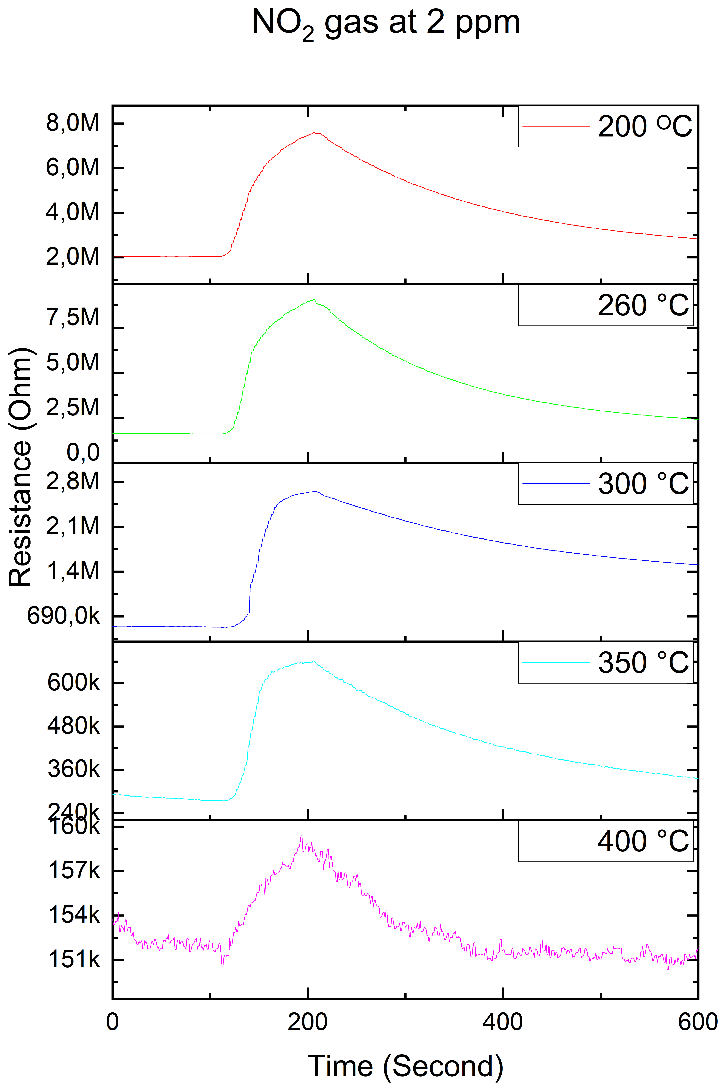
**

**Figure S6.** Raw data used for the sensitivity graphs in Figure 5a in the main draft (**left**) BFO – response to NO_2_ gas (**right**) BSFO response to NO_2_ gas.
